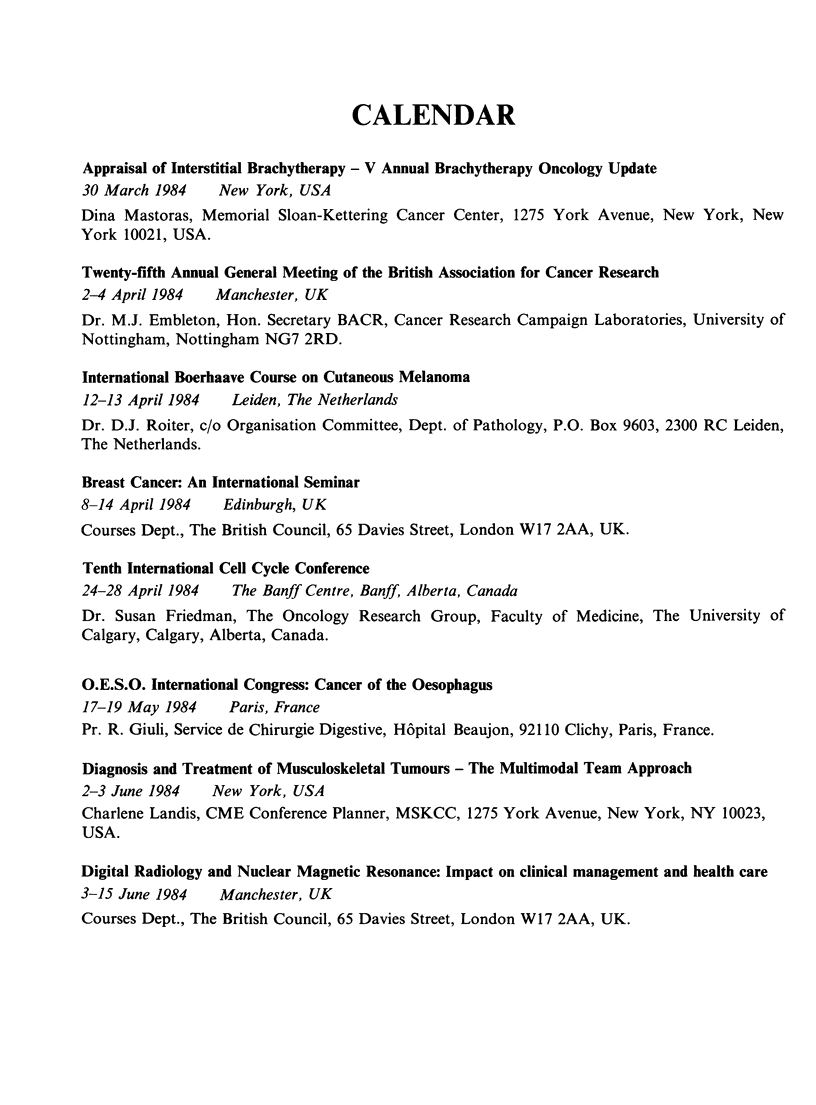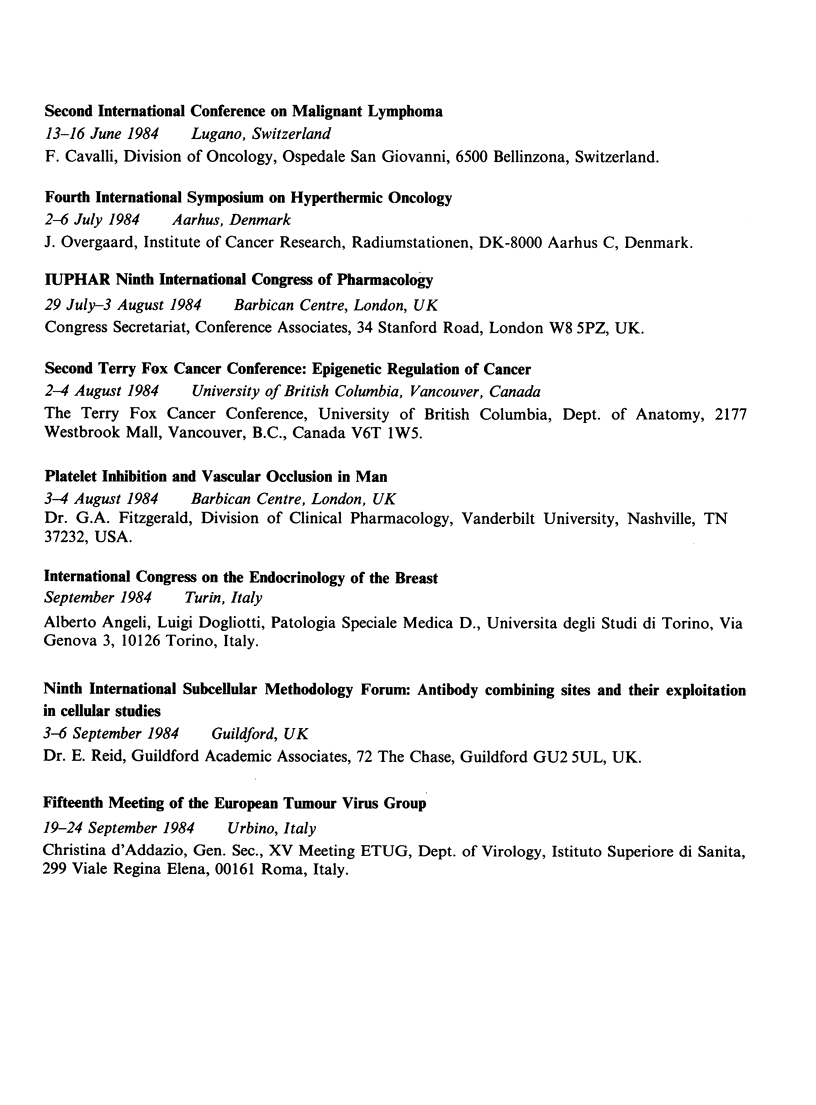# Calendar

**Published:** 1984-03

**Authors:** 


### CALENDAR

**Appraisal of Interstitial Brachytherapy – V Annual Brachytherapy Oncology Update**
*30 March 1984    New York, USA*
Dina Mastoras, Memorial Sloan-Kettering Cancer Center, 1275 York Avenue, New York, New York 10021, USA.

**Twenty-fifth Annual General Meeting of the British Association for Cancer Research**
*2–4 April 1984    Manchester, UK*
Dr. M.J. Embleton, Hon. Secretary BACR, Cancer Research Campaign Laboratories, University of Nottingham, Nottingham NG7 2RD.

**International Boerhaave Course on Cutaneous Melanoma**
*12–13 April 1984    Leiden, The Netherlands*
Dr. D.J. Roiter, c/o Organisation Committee, Dept. of Pathology, P.O. Box 9603, 2300 RC Leiden, The Netherlands.

**Breast Cancer: An International Seminar**
*8–14 April 1984    Edinburgh, UK*
Courses Dept., The British Council, 65 Davies Street, London W17 2AA, UK.

**Tenth International Cell Cycle Conference**
*24–28 April 1984    The Banff Centre, Banff, Alberta, Canada*
Dr. Susan Friedman, The Oncology Research Group, Faculty of Medicine, The University of Calgary, Calgary, Alberta, Canada.

**O.E.S.O. International Congress: Cancer of the Oesophagus**
*17–19 May 1984    Paris, France*
Pr. R. Giuli, Service de Chirurgie Digestive, Hôpital Beaujon, 92110 Clichy, Paris, France.

**Diagnosis and Treatment of Musculoskeletal Tumours – The Multimodal Team Approach**
*2–3 June 1984    New York, USA*
Charlene Landis, CME Conference Planner, MSKCC, 1275 York Avenue, New York, NY 10023, USA.

**Digital Radiology and Nuclear Magnetic Resonance: Impact on clinical management and health care**
*3–15 June 1984    Manchester, UK*
Courses Dept., The British Council, 65 Davies Street, London W17 2AA, UK.

**Second International Conference on Malignant Lymphoma**

*13–16 June 1984* *Lugano, Switzerland*

F. Cavalli, Division of Oncology, Ospedale San Giovanni, 6500 Bellinzona, Switzerland.

**Fourth International Symposium on Hyperthermic Oncology**

*2–6 July 1984* *Aarhus, Denmark*

J. Overgaard, Institute of Cancer Research, Radiumstationen, DK-8000 Aarhus C, Denmark.

**IUPHAR Ninth International Congress of Pharmacology**

*29 July–3 August 1984* *Barbican Centre, London, UK*

Congress Secretariat, Conference Associates, 34 Stanford Road, London W8 5PZ, UK.

**Second Terry Fox Cancer Conference: Epigenetic Regulation of Cancer**

*2–4 August 1984* *University of British Columbia, Vancouver, Canada*

The Terry Fox Cancer Conference, University of British Columbia, Dept. of Anatomy, 2177 Westbrook Mall, Vancouver, B.C., Canada V6T 1W5.

**Platelet Inhibition and Vascular Occlusion in Man**

*3–4 August 1984* *Barbican Centre, London, UK*

Dr. G.A. Fitzgerald, Division of Clinical Pharmacology, Vanderbilt University, Nashville, TN 37232, USA.

**International Congress on the Endocrinology of the Breast**

*September 1984* *Turin, Italy*

Alberto Angeli, Luigi Dogliotti, Patologia Speciale Medica D., Universita degli Studi di Torino, Via Genova 3, 10126 Torino, Italy.

**Ninth International Subcellular Methodology Forum: Antibody combining sites and their exploitation in cellular studies**

*3–6 September 1984* *Guildford, UK*

Dr. E. Reid, Guildford Academic Associates, 72 The Chase, Guildford GU2 5UL, UK.

**Fifteenth Meeting of the European Tumour Virus Group**

*19–24 September 1984* *Urbino, Italy*

Christina d'Addazio, Gen. Sec., XV Meeting ETUG, Dept. of Virology, Istituto Superiore di Sanita, 299 Viale Regina Elena, 00161 Roma, Italy.